# Targeted EV to Deliver Chemotherapy to Treat Triple-Negative Breast Cancers

**DOI:** 10.3390/pharmaceutics14010146

**Published:** 2022-01-07

**Authors:** Yingnan Si, Kai Chen, Hanh Giai Ngo, Jia Shiung Guan, Angela Totoro, Zhuoxin Zhou, Seulhee Kim, Taehyun Kim, Lufang Zhou, Xiaoguang Liu

**Affiliations:** 1 Department of Biomedical Engineering, University of Alabama at Birmingham (UAB), 1825 University Blvd, Birmingham, AL 35294, USA; yingnan@uab.edu (Y.S.); kaisdzb@uab.edu (K.C.); hanh96@uab.edu (H.G.N.); angmshel@uab.edu (A.T.); zhouzhx@uab.edu (Z.Z.); lfzhou@uab.edu (L.Z.); 2 Department of Medicine, University of Alabama at Birmingham (UAB), 703 19th Street South, Birmingham, AL 35294, USA; guan0926@uab.edu (J.S.G.); seulheekim@uabmc.edu (S.K.); kimth@uab.edu (T.K.)

**Keywords:** triple-negative breast cancers, EGFR/CD47 targeting, extracellular vesicle, verrucarin A

## Abstract

Triple-negative breast cancers (TNBCs) are heterogeneous and metastatic, and targeted therapy is highly needed for TNBC treatment. Recent studies showed that extracellular vesicles (EV) have great potential to deliver therapies to treat cancers. This study aimed to develop and evaluate a natural compound, verrucarin A (Ver-A), delivered by targeted EV, to treat TNBC. First, the surface expression of epidermal growth factor receptor (EGFR) and CD47 were confirmed with immunohistochemistry (IHC) staining of patient tissue microarray, flow cytometry and Western blotting. EVs were isolated from HEK 293F culture and surface tagged with anti-EGFR/CD47 mAbs to construct mAb-EV. The flow cytometry, confocal imaging and live-animal In Vivo Imaging System (IVIS) demonstrated that mAb-EV could effectively target TNBC and deliver the drug. The drug Ver-A, with dosage-dependent high cytotoxicity to TNBC cells, was packed in mAb-EV. The anti-TNBC efficacy study showed that Ver-A blocked tumor growth in both 4T1 xenografted immunocompetent mouse models and TNBC patient-derived xenograft models with minimal side effects. This study demonstrated that the targeted mAb-EV-Ver-A had great potential to treat TNBCs.

## 1. Introduction

Triple-negative breast cancers (TNBCs) are HER2^−^/ER^−^/PR^−^ and highly aggressive, metastatic, and heterogeneous (five subtypes, revised algorithm TNBCtype-IM) [1,2,3]. The standard chemotherapies such as anthracycline-taxane are the main treatment strategy, but TNBCs often regrow with recurrence rate of >50% and poor survival after primary treatment [4,5,6,7]. Recently, the combined immunotherapy-chemotherapy, Atezolizumab (immune checkpoint inibitor) and Abraxane (nab-paclitaxel), has been developed to treat PD-L1^+^ TNBC [8,9,10]. The antibody-drug conjugate, Sacituzumab Govitecan, has been approved to treat trophoblast cell-surface antigen 2 (Trop-2)^+^ TNBC [11,12,13,14,15]. The success of these therapeutics demonstrated that targeted therapy or therapeutic combination has great potential to treat TNBCs. Considering the challenges of early metastases and relapses [16], heterogeneity of TNBCs, new targeted therapies are highly desired.

Extracellular nanovesicles (EVs) are natural nanoparticles that are secreted by cells to facilitate the communication among cells through transporting endogenous RNA, DNA, and proteins [17,18]. More importantly, EV has great potential to deliver therapies for cancer treatment [17,18,19,20,21,22] due to its advantages of low immunogenicity and enhanced circulation retention. For instance, the human dendritic cell-secreted EVs can activate the immunity of T and NK cells [23,24,25]. To achieve tumor-specific targeting by EV, cell engineering technologies have been applied to surface display various peptides such as iRGD, RVG, EGFR-GE11, or HER2/neu [19,21,26,27]. Our previous studies have established a platform to produce cancer-targeted EV using a stirred-tank bioreactor and monoclonal antibody (mAb) surface tagging technology [28,29]. Furthermore, we have developed a dual-targeted EV to cover more patients than in the previous study [29]. 

It has been reported that the EGFR receptor, which enhances DNA replication [30,31,32,33] and stimulates cancer cell proliferation via phosphorylating phosphatidylinositol 3-kinase (PI3K) [34,35], is overexpressed in over 52% of TNBC patients [36,37], 40% of lung cancer [38,39], and over 80% of head and neck cancers [36,37,40]. The glycoprotein CD47, which provides a “don’t eat me” signal via signal regulatory protein alpha (SIRPα) [41,42], is overexpressed on the surface of TNBC cells. The expression of CD47 can be upregulated by standard chemotherapies such as gemcitabine and carboplatin [43,44,45], indicating that CD47 is an ideal target to treat recurrent or drug-resistant TNBCs. In this study, we confirmed the overexpression of EGFR and CD47 receptors in TNBC patient tissues and constructed anti-EGFR/CD47 mAb tagged EV as a drug delivery vehicle. 

We have recently identified a natural compound, Verrucarin A (Ver-A), and demonstrated a high anti-cancer cytotoxicity to treat neuroendocrine tumors in our previous study [29]. Ver-A was originally isolated from the metabolites of *Myrothecium verrucaria* cultivated in saltwater culture [46]. The literature has reported various anti-proliferation and pro-apoptosis mechanisms in breast cancer [47] and other cancers [48,49]. For instance, Ver-A can arrest cells in the synthesis phase, inhibit cell cycle via regulatory proteins such as cyclin D1, cyclin E and cyclin-dependent kinases, induce apoptosis by inhibiting Bcl-2 family proteins, cause mitochondrial inner membrane potential depolarization, or downregulate the signaling proteins such as pro-survival phospho-Akt (p-Akt), nuclear factor κB (NF-κB) and mammalian target of rapamycin (p-mTOR) [50]. Therefore, we evaluated the potential of the natural drug Ver-A to treat TNBCs. 

The objective of this study was to develop and evaluate a new targeted therapy to treat the highly aggressive TNBCs. Specifically, we constructed an EGFR/CD47 dual-targeted mAb-EV to pack the highly potent Ver-A. The TNBC-targeting and anti-tumor efficacy of mAb-EV-Ver-A were evaluated in both immunocompetent xenograft models and patient-derived xenograft (PDX) models. The results demonstrated that mAb-EV-Ver-A had great potential to target and treat TNBCs. 

## 2. Materials and Methods

### 2.1. Cell Lines and Media

Multiple human TNBC cell lines, including MDA-MB-468, MDA-MB-231, BT-20 (ATCC, Manassas, VA, USA) and MDA-MB-231-FLuc (GenTarget, San Diego, CA, USA), and mouse TNBC cell line 4T1-Luc (ATCC) were used to in vitro evaluate the TNBC targeting and cytotoxicity of the developed dual-targeted therapeutics. The human TNBC cells were maintained in a DMEM/F12 medium supplemented with 4 g/L of glucose, 4 mM of L-glutamine, and 10% of fetal bovine serum (FBS) in T25 or T75-flasks. The mouse TNBC 4T1 cells were maintained in RPMI medium supplemented with the same levels of glucose, L-glutamine, and FBS as DMEM/F12. The hybridoma cells for anti-CD47 mAb production were adapted in Hybridoma-SFM (serum free medium) and maintained in shaker flasks with an agitation (Agt) speed of 130 rpm. All cell cultures were incubated at 37 °C and 5% CO_2_ in a humidified incubator (Caron, Marietta, OH, USA). All media, supplements, bioreagents and kits were purchased from Fisher Scientific (Waltham, MA, USA) unless otherwise specified. 

### 2.2. Patient Tissue Microarray (TMA) and Immunohistochemistry (IHC) Staining

To evaluate the expression of surface receptors, IHC staining [51] of the patient TMA containing 150 cores and 50 cases of ER^−^/PR^−^/HER2^−^ breast carcinoma (US Biomax, Derwood, MD, USA) was performed with anti-EGFR antibody and anti-CD47 antibody, respectively. Specficially, the TMA slides were baked overnight at 60 °C, then de-paraffinized in xylene and hydrated with ethanol and deionized water. The tissue sections were subjected to antigen retrieval by 0.01 M Tris-1 mM EDTA buffer (pH 9) for EGFR stain and by 0.01 M sodium citrate buffer (pH 6) for CD47 stain in a pressure cooker for 5 min. After being washed gently in deionized water, the TMA slides were transferred into 0.05 M Tris buffer with 0.15 M NaCl and 0.1% *v*/*v* Triton-X-100 (TBST, pH 7.6). The endogenous peroxidase was blocked with 3% hydrogen peroxide for 15 min and slides were incubated with 5% normal goat serum for 45 min at room temperature to reduce further nonspecific background staining. All slides were then incubated at 4 °C overnight with anti-EGFR (Abcam, Cambridge, UK, ab52894, Rabbit monoclonal, 1/100 dilution) or anti-CD47 (Abcam, ab175388, Rabbit polyclonal, 1/100 dilution). After washing with TBST (Tris-Buffered Saline, 0.05% Tween20), sections were incubated with the Goat Anti-Rabbit IgG H&L secondary antibody conjugated with HRP (Abcam ab6721, 1:1000.) The ImmPACT DAB Peroxidase (HRP) Substrate Kit was used as the chromogen and hematoxylin (Richard-Allen Scientific, Kalamazoo, MI, USA) as the counterstain.

### 2.3. Anti-CD47 mAb Production

We developed the anti-CD47 mAb using hybridoma technology and produced and evaluated mAb following our published procedure [52,53]. Briefly, the anti-CD47 mAb (IgG2b/kappa) was produced using hybridoma culture at Temp 37 °C and Agt of 110 rpm, which was fed with 4 g/L of glucose, 4 mM of L-glutamine, and 3.5 g/L Cell Boost #6 on Day 3. The mAb purification was performed using a liquid chromatography system (Bio-Rad, Hercules, CA, USA) equipped with Bio-Scale Mini UNOsphere SUPrA column.

### 2.4. Construction of mAb-EV-Ver-A, -Cy5 or -Cy7

The mAb-EV-Ver-A was generated following our previously established protocols [28,29]. First, EV was produced using HEK 293F cell culture in FreeStyle^TM^ 293 expression medium at Temp 37 °C, pH 7.0 and Agt 70 rpm, purified using Vivaspin 300 kDa MWCO concentrator, and stored in PBS containing 25 mM trehalose at −80 °C for up to 6 months. Second, the anti-human EGFR and CD47 mAbs were tagged to EV via DSPE-PEG-NHS linker to generate mAb-EV, which was further modified with mPEG-DSPE to improve its circulation stability. The mAb-EV was titrated using NanoSight (Malvern Panalytical, Malvern, UK) with SCMOS camera (5 captures per sample), a pump speed of 50 and a detection threshold of 5. Third, the anti-cancer payload Ver-A was loaded to mAb-EV via incubation using formulation of 1 × 10^11^ EV and 0.101 mg (200 nmol) of Ver-A in 8.6 mL of PBS. The mAb-EV was labelled with fluorescent dye (i.e., Cy5 or Cy7) to investigate TNBC targeting or drug release by mixing 1 × 10^12^ EV and 16.7 nmol of dye. The unpacked free drug or unlabeled free dye was removed using Vivaspin 100 kDa column. The drug loading capability was titrated using HPLC (Shimadzu, Columbia, MD) equipped with C18 column using mobile phase of 5−90% CH_3_CN/H_2_O containing 0.1% trifluoroacetic acid following the protocol reported in literature [54], but we found that EV particle changed the HPLC titration accuracy. The in vitro anti-cancer cytotoxicity assay of mAb-EV-Ver-A and free Ver-A (control) showed the functional loading rate of Ver-A was ~50%. 

### 2.5. In Vitro Anti-TNBC Cytotoxicity Assay 

The anti-TNBC cytotoxicity assay was carried out in 96-well plates using MDA-MB-231 and MDA-MB-468 cells with seeding density of 0.5 × 10^5^ cells/mL in 200 µL of DMEM/F12 complete medium with triplication. Multiple dosages of standard chemotherapy GC (0–500 nM, control) or Ver-A (0–8 nM) were tested in a three-day assay. The cytotoxicity was measured using CellTiter-Glo Luminescent Cell Viability Assay (Promega, Madison, MI, USA) and described as relative viability. The IC_50_ value was calculated using ED50V10 Excel add-in.

### 2.6. Western Blotting 

The TNBC cells were lysed with Thermo Scientific M-PER Mammalian Protein Extraction Reagent and the lysate protein concentrations were titrated with a BCA Protein Assay Kit. The protein samples (30 µg) were loaded to a non-reducing Bolt™ 4 to 12% Bis-Tris Mini Protein Gel, electro-transferred to PVDF membrane using PowerEase^TM^ Touch 350 W Power Supply, and blocked with 1× TBS, 5% dry skim milk and 0.01% Tween-20 for 1 h at room temperature. The target proteins were detected with 1:1000 diluted primary rabbit anti-human antibody overnight at 4 °C, followed by incubating with 1:3000 diluted HRP-conjugated secondary mouse anti-rabbit antibody. The target proteins were detected using Luminata Forte Western HRP substrate (Millipore, Boston, MA, USA). The Western blots were imaged with MyECL imager and quantified using ImageJ software (NIH, Bethesda, MD, USA). 

### 2.7. Flow Cytometry

The surface binding of anti-EGFR mAb and CD47 mAb to TNBC cells lines (MDA-MB-231, MDA-MB-468, and BT-20) was analyzed using a BD LSRII flow cytometer (BD Biosciences, San Jose, CA, USA). Briefly, 1 µg of mAb that was labelled with an Alexa Fluor™ antibody 647 labelling kit (Life Tech, part of Fisher, Carlsbad, CA, USA) was mixed with one million cells at room temperature for 30 min [29,55]. The AF647 signal was detected with Alexa Fluor 700 laser and data were analyzed using FlowJo V5.0 (TreeStar, Inc., Ashland, OR, USA).

### 2.8. Live-Cell Confocal Imaging 

The live-cell confocal microscopy imaging was collected to confirm the targeting and internalization of mAb-EV following our established protocol [29,56]. The TNBC (MDA-MB-468) cells were cultivated on 15-mm glass-bottom dish and transfected with BacMam GFP virus to express eGFP in cytoplasm for 24 hrs. Then 33 µg of EGFR mAb-EV-Cy5 or 33 µg of CD47 mAb-EV-Cy5 was added to culture and incubated for 24 hrs. Finally, the eGFP and Cy5 was imaged using a Nikon A1R-HD25 confocal microscope with a high-speed resonance scanner (Nikon USA, Melville, NY, USA) with the 489 and 561 laser lines, respectively. The confocal images were processed offline using ImageJ software (National Institutes of Health, Bethesda, MD, USA) for co-localization analysis. 

### 2.9. In Vivo Imaging System (IVIS) Imaging 

The animal study protocol (IACUC-22200) was approved by the Institutional Biosafety Committee of University of Alabama at Birmingham on 11 January 2021. About 3 × 10^6^ cells of human TNBC (MDA-MB-231-FLuc) were injected into the fourth mammary fat pad of the NSG (NOD scid gamma) mice. Mice will develop tumors within 2 weeks post cells injection. When tumor volume reached 50–100 mm^3^, 10 × 10^10^ mAbs-EV-Cy7 (construction details see Section 2.4) was intravenously (i.v.) administrated via tail vein for TNBC-targeting analysis. At 24 hrs post mAb-EV-Cy7 injection, mice were imaged under IVIS Lumina Series III (PerkinElmer, Waltham, MA, USA) with wavelength of 660 nm/710 nm (excitation/emission) and exposure time of 3 s. The TNBC targeting was confirmed by the overlap of bioluminescence (FLuc) and fluorescence (Cy7). In addition, the TNBC tumor and major organs (lung, heart, spleen, liver, and kidney) were extracted to collect ex vivo images to detect possible off-target side effects. 

### 2.10. Primary TNBC Xenograft Models and In Vivo Treatment

The primary TNBC xenograft mouse models were generated by injecting 1 × 10^6^ cells of 4T1-FLuc cells into the fourth mammary fat pad of the six-week-old BALB/cJ female mice (Jackson Labs, Bar Harbor, ME, USA) [52,53,56]. Mice were randomized into eight groups (*n* = 5–6) when tumor volume reached ~100 mm^3^ within 10–14 days post cells implantation. These models were used to analyze the possible toxicity, evaluate the dosage effect of mAb-EV-Ver-A and GC, and test the anti-TNBC efficacy of the dual-targeted therapy. Specifically, the TNBC xenograft mice were treated with 2 mg/kg-BW of GC with i.p. injection daily for the first six days. Then mice were treated with five dosages of EGFR/CD47 mAb-EV-Ver-A (i.e., 0, 0.5, 1.5, 2, and 2.5 mg/kg), 0.5 mg/kg of EGFR mAb-EV-Ver-A, 0.5 mg/kg of CD47 mAb-EV-Ver-A, and mAb-EV via i.v. injection on a Q3D × 4 schedule (i.e., three-day interval for four injections). Tumor volume was measured using an electronic caliper and calculated as “width × width × length/2”. The body weight data were collected every other day. The in vivo treatment study was ended when tumor volume reached >1000 mm^3^ in the control (PBS) group. At the end of the experiment, mice were euthanized and sacrificed to collect TNBC tumor and major organs for H&E staining to evaluate the possible toxicity. 

### 2.11. TNBC Patient-Derived Xenograft (PDX) Models and In Vivo Treatment

The TNBC PDX xenograft donor mice were purchased from The Jackson Laboratory and maintained at low passages of 2–4 in host NSG mice following our established protocol [57]. Briefly, either fresh harvested tumor or fresh frozen tumor tissues were minced into small fragments (<1 mm^3^) and subcutaneously (s.c.) injected into the right flank of NSG mouse. After 100 mm^3^ of tumor xenograft was detected, mice were randomized into two groups and treated with 2 mg/kg GC via i.p. injection for six days followed with mAb-EV or 4 mg/kg mAb-EV-Ver-A via tail vein injection on a Q3D × six schedule (*n* = 5). Tumor volume was measured by a caliper every two or three days and tumor volumes were calculated as (width × width × length)/2 in millimeter with end point of volume > 1000 mm^3^. 

### 2.12. Hematoxylin and Eosin (H&E) Staining

The tumor tissue and/or important organs (lung, heart, spleen, liver, kidney) were harvested, dehydrated in ethanol, cleared in xylene, embedded in paraffin, sectioned at 5 μm with Leica microtome, and mounted on frosted microscope slides in University Pathology Core. The paraffin sectioned slides were dewaxed with xylene and gradient hydrated with 100%–50% ETOH and dH_2_O. The hydrated slides were stained with hematoxylin, rinsed with deionized water, dipped in 1% HCl in 70% ETOH, immersed in 1% NH_4_OH for blue color development overnight, and stained with eosin for 30 secs. The stained slides were dehydrated in 95% and 100% ethanol and cleared in xylene. 

### 2.13. Statistical Analysis

All experimental data were expressed as mean ± standard error of the mean (SEM). The significance of differences among groups was analyzed using a one-way ANOVA followed by post-hoc (Dunnett’s) analysis. Statistical analysis was performed using GraphPad Prism and *p*-values of <0.05 were considered to be significant.

## 3. Results

The relevant literature, in addition to [36,37,43,44,57] this study, revealed high expression of the surface receptors EGFR and CD47 in TNBC patient tissues. This study constructed an EGFR/CD47 dual-targeted mAb-EV to deliver the natural compound Ver-A for TNBC treatment. The anti-tumor efficacy was confirmed in both mouse TNBC xenograft immunocompetent models and in a PDX model. 

### 3.1. EGFR and CD47 Expression in TNBCs

**Figure 1:**
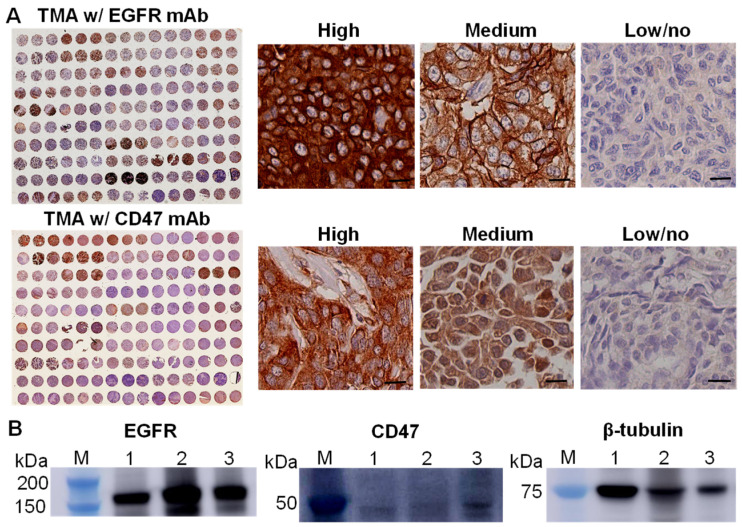
Surface receptors of EGFR and CD47 in TNBCs. (**A**) IHC staining of TNBC TMA with EGFR and CD47 antibody and the representative images of high, medium and low/no expression of EGFR and CD47. Case of 50 and 150 cores. Scale bar equals 20 µm. (**B**) Western blotting analysis of EGFR, CD47 and ꞵ-tubulin in MDA-MB-231 (Lane 1), MDA-MB-468 (Lane 2) and BT-20 (Lane 3).

The surface expressions of EGFR and CD47 in TNBCs were assessed using the IHC staining of patient tissue microarray (150 cores and 50 cases, *n* = 3) with anti-EGFR antibodies and anti-CD47 antibodies, respectively. The images of whole TMA slide and the representative images of the tissues with high, medium and low/no expression of surface receptor EGFR and CD47 were presented in Figure 1A. The Image J analysis and quantification showed that 56% and 69% of TNBC tissues had high or medium level of EGFR and CD47, respectively. Western blotting analysis confirmed the expression of EGFR and CD47 in MDA-MB-231, MDA-MB-468 and BT20 cell lines (Figure 1B). It is found that EGFR has higher expression than CD47 in TNBC cells. 

### 3.2. Construction of Dual-Targeted mAb-EV-Ver-A

**Figure 2:**
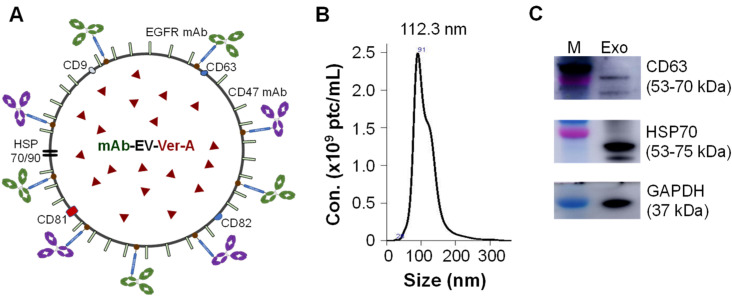
Construction and characterization of dual-targeted mAb-EV-drug. (**A**) Structure of anti-EGFR/CD47 mAb-EV-Ver-A. (**B**) Size distribution analysis by NanoSight. (**C**) Western blotting of EV biomarkers (CD63, HSP70, GAPDH).

The EGFR/CD47 mAb-Exo-Ver-A was constructed following our previously developed platform, i.e., stirred-tank bioreactor-based EV production and mAb surface tagging technology [29], with optimization (Figure 2A). Briefly, EVs were generated from HEK293F cells in serum free medium, purified with 300 kDa size exclusion column, and surface tagged with anti-EGFR mAb (Cetuximab) and our anti-CD47 via NHS-PEG-DSPE [45]. The mPEG-DSPE stabilizer was used to modify EV membrane to improve the circulation stability [28]. NanoSight analysis showed EV particle size distribution of 78.2–151.1 nm with mean size of 112.3 ± 1.5 nm (Figure 2B). Western blotting analysis confirmed the surface biomarkers of CD63 and HSP70 (Figure 2C). 

### 3.3. TNBC Targeting by EGFR/CD47 mAb-EV

**Figure 3:**
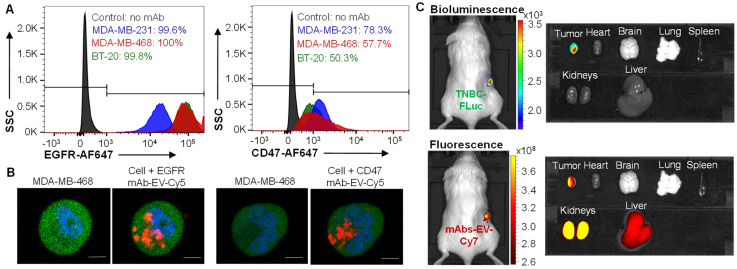
Evaluations of in vitro surface binding, internalization and in vivo TNBC targeting of mAb-EV. (**A**) Flow cytometry analysis of surface binding of anti-EGFR mAb and anti-CD47 mAb to TNBC cells (MDA-MB-231, MDA-MB-468 and BT-20). (**B**) Live-cell confocal microscopy imaging of internalization of EGFR mAb-EV-Cy5 and CD47 mAb-EV-Cy5. Cytoplasm of TNBC cells was labeled with GFP (green) and mAb-EV was labeled with fluorescent dye Cy5 (red). Scale bar equals 10 µm. (**C**) Live-animal and ex vivo IVIS imaging to confirm TNBC targeting of EGFR/CD47 mAb-Cy7 at 24 hr post tail vein injection.

Flow cytometry analysis was performed to evaluate the surface binding of EGFR and CD47 mAbs in TNBC cell lines, including MDA-MB-231, MDA-MB-468 and BT20, with staining conditions of 1 µg mAb-AF647 per million cells at room temperature. The results showed that EGFR mAb had a surface binding rate of 99.6%, 100% and 99.8%, and CD47 mAb had surface binding rate of 78.3%, 57.7% and 50.3% to MDA-MB-231, MDA-MB-468 and BT20, respectively (Figure 3A). The lower surface binding of CD47 mAb than EGFR mAb was consistent with the result of the lower surface expression of CD47 as presented in Figure 1B. Furthermore, mAb-EV was labeled with fluorescent dye Cy5 to test its TNBC targeting and drug delivery capability (Figure 3B). The live-cell confocal microscopy imaging showed that both EGFR mAb-EV and CD47 mAb-EV (displayed as red) internalized into the cytoplasm (displayed as green) of MDA-MB-468 cells, demonstrating its effective drug delivery capability. 

It is found that EGFR mAb and CD47 mAb target human and mouse TNBC cells in our previous studies [56], so the immunocompromised TNBC xenograft mouse models were used to evaluate TNBC targeting in vivo. In this study, the MDA-MB-231-FLuc xenograft NSG model was i.v. injected with dual-targeted EGFR/CD47 mAb-EV-Cy7. Live-animal IVIS imaging revealed that mAb-EV (indicated by Cy7 fluorescence) targeted and accumulated in TNBC tumor (indicated by FLuc bioluminescence) at 24 h (Figure 3C). Ex vivo imaging confirmed that there was no obvious off-targeting of mAb-EV in major organs such as the brain, heart, lung and spleen. As consistent with the literature [17,18,19,20,21,22], Cy7 was detected in the liver and kidney. Altogether, the designed EGFR/CD47 mAb-EV can target TNBC and effectively deliver payloads. 

### 3.4. In Vitro Anti-TNBC Cytotoxicity

**Figure 4:**
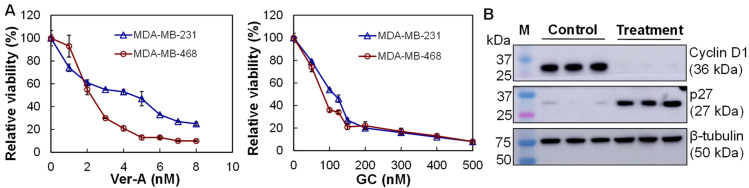
In vitro anti-TNBC cytotoxicity of Ver-A. (**A**) IC_50_ curves of Ver-A in TNBC cells with GC as control. ○: MDA-MB-468, and Δ: MDA-MB-231. (**B**) Western blotting analysis of proliferation biomarkers (cyclin D1, P27, ꞵ-tubulin) post EV-Ver-A treatment with PBS as control. Data represent mean ± SEM, *n* = 3.

The in vitro anti-TNBC cytotoxicity of Ver-A and GC (standard chemotherapy, control) was evaluated in TNBC MDA-MB-231 and MDA-MB-468 cells to collect IC_50_ curves. The Ver-A cytotoxicity assay showed that the relative viabilities were 100%–25% in MDA-MB-231 and 100%–10% in MDA-MB-231 cells at dosages of 0–8 nM, respectively (Figure 4A). The GC cytotoxicity assay showed relative viabilities of 100%–8% in MDA-MB-231 and 100%–8% in MDA-MB-468 at dosages of 0–500 nM (Figure 4A). The calculated IC_50_ values were 82–114 nM for GC and 2–4 nM for Ver-A in these two treated TNBC cells. These data showed that the natural compound Ver-A was more efficient to treat TNBC than GC. Western blotting of MDA-MB-468 cells treated with 5 nM of Ver-A demonstrated that the expressions of proliferation protein cyclin D1 was significantly reduced and expression of cyclin dependent kinase inhibitor p27 was increased at 48 hrs post treatment (Figure 4B). 

### 3.5. Tolerated Dosage (TD) and Anti-Tumor Efficacy in Immunocompetet Models

**Figure 5:**
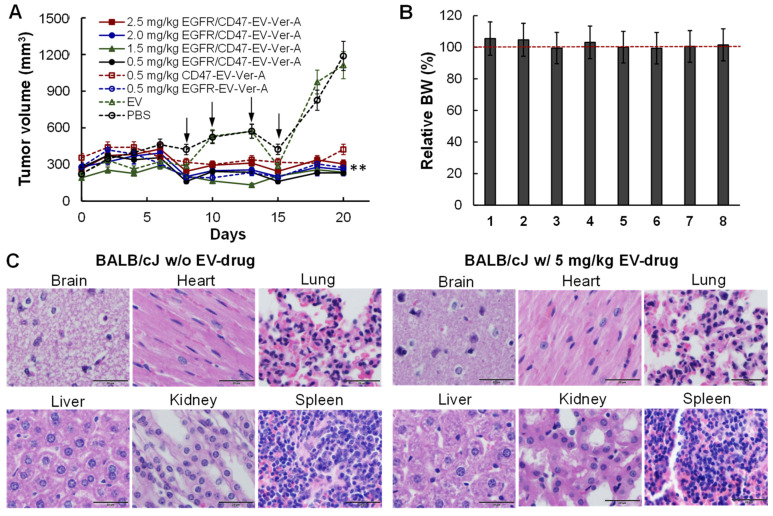
In vivo anti-TNBC efficacy in 4T1 xenografted BALB/cJ mice. (**A**) Profile of tumor volume in xenograft models that were treated with targeting delivered Ver-A. Four dosages of EGFR/CD47 mAbs-EV-Ver-A (0, 0.5, 1.5, 2 and 2.5 mg/kg) and three controls (EGFR mAb-EV-Ver-A, CD47 mAb-EV-Ver-A, and mAbs-EV) were *i.v.* injected on Days 8, 10, 13 and 15. ** *p* ≤ 0.05. Data represent mean ± SEM, *n* = 5–6. (**B**) Body weight change of mice in groups of PBS, EV, 0.5 mg/kg of EGFR mAb-EV-Ver-A, 0.5 mg/kg of CD47 mAb-EV-Ver-A, 0.5, 1.5, 2.0 and 2.5 mg/kg of EGFR/CD47 mAb-EV-ver-A. (**C**) H&E staining of important organs, including brain, heart, lung, liver, kidney, and spleen, of mouse in 2.5 mg/kg EGFR/CD47 mAb-EV-Ver-A treatment group. Scale bar equals to 20 µm.

Both TD and anti-tumor studies in mouse TNBC (4T1-FLuc) were evaluated in xenograft BALB/cJ models. When tumor volumes reached >250 mm^3^, mice were treated with 2 mg/kg-BW of GC with i.p. injection daily for 6 days in the treatment groups. Total of four dosages of EGFR/CD47 mAb-EV-Ver-A (i.e., 0.5, 1.5, 2.0, 2.5 mg/kg) and 0.5 mg/kg of EGFR mAb-EV-Ver-A and 0.5 mg/kg of CD47 mAb-EV-Ver-A were i.v. injected via tail vein on Days eight, 10, 13 and 15 with PBS and EV as controls (free drug was not tested in vivo). The tumor volume profiles showed that mAb-EV-Ver-A effectively inhibited TNBC tumor growth while EV had no effect (Figure 5A). The increase of Ver-A dosage to 2.5 mg/kg had similar anti-tumor efficacy as 0.5 mg/kg, indicating that Ver-A could not kill all TNBC cells in vivo and combination with other therapy is needed to eliminate TNBC tumors. In addition, dual targeting did not show obvious advantages other than single targeting in 21-day treatment. The change of mouse body weight in all groups was in the range of 5–10% (Figure 5B), and no obvious changes in water intake, breathing, locomotion, and survival were observed. When tumor volume reached 1000 mm^3^, mice were sacrificed to collect the brain, heart, lung, liver, kidney and spleen for sectioning and H&E staining. None of these organs in mice treated with 2.5 mg/kg EGFR/CD47 mAb-EV-Ver-A had any obvious morphology change or necrosis compared to PBS group (Figure 5C). These results indicated that the mAb-EV-delivered Ver-A had no evident off-target effects in vivo, and the potential toxicity was minimal, consistent with the TNBC-targeting results observed in IVIS imaging. 

### 3.6. In Vivo Anti-Tumor Efficacy in PDX Models

**Figure 6:**
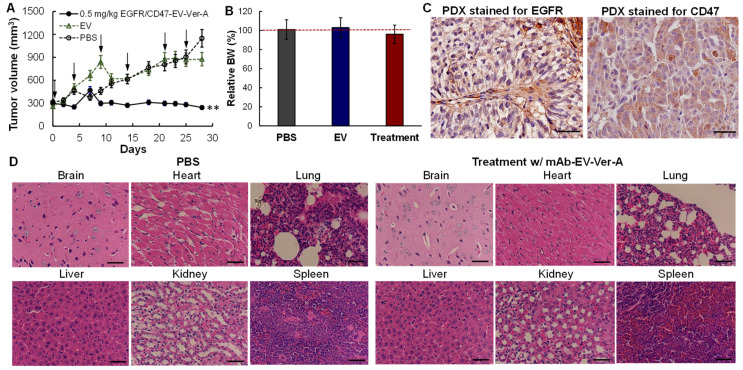
In vivo anti-tumor efficacy of mAb-EV-Ver-A in TNBC PDX models. (**A**) Tumor volume post treatment (*n* = 5). Arrow indicates the i.v. injection of PBS, EV and mAb-EV-Ver-A on Days 0, 4, 9, 15, 21 and 26. ** *p* < 0.05 vs. PBS using ANOVA followed by Dunnett’s *t*-test. (**B**) The relative body weight after treatment. (**C**) IHC staining of PDX xenograft tissues showing positive expression of EGFR and CD47. Scale bar equals to 100 nm. (**D**) H&E staining of important organs. Scale bar equals to 40 µm.

Patient-derived xenograft (PDX) models can capture the heterogeneity and microenvironment of TNBC and maintain the biological behavior of patient tumors, which is important in in vivo evaluations of the developed new therapy. We have successfully passaged and propagated TNBC PDX xenograft (JAX, J000103634) in NSG female mice. The established PDX models were treated with PBS (negative control), EV (vehicle control), or 0.5 mg/kg mAb-EV-Ver-A on a Q4D × six schedule (*n* = 5), i.e., Days 0, 4, 9, 15, 21 and 26. The tumor volume and mouse body weight of PDX models were monitored twice a week. As described in Figure 6A, Ver-A delivered with mAb-EV effectively inhibited the PDX tumor growth, similar to the results in immunocompetent models (Figure 5A). The changes of mouse body weight in all eight control or treatment groups were less than 10% (Figure 6B). IHC staining of PDX tissue section confirmed the high surface expression of EGFR and CD47 (Figure 6C). In addition, the pathologic assessment of H&E-stained important organs, including brain, heart, lung, liver, kidney and spleen, did not show any signs of acute or chronic inflammation or apoptotic or necrotic regions in the PBS control group and the mAb-EV-Ver-A treatment group (Figure 6D). These data indicated that the targeting delivered Ver-A can effectively treat TNBC with minimal toxicity. 

## 4. Discussion

The high heterogeneity of HER2^−^/ER^−^/PR^−^ TNBCs and lack of suitable targeting receptors are the major challenges to develop targeted therapy for TNBC treatment. This study and/or previous studies [56] revealed that EGFR is overexpressed in 56% TNBC patient tissues and CD47 is overexpressed in 69% of TNBC patient tissues. The tumor-targeting analysis confirmed both EGFR and CD47 are good targets of TNBC. Moreover, the CD47 expression is up-regulated by chemotherapy [43,44] and it could be an ideal candidate target in drug-resistant or recurrent TNBC. Therefore, the EGFR/CD47 dual-targeted drug delivery vehicle, i.e., mAb-EV constructed in this study, has multiple advantages: (1) it covers most TNBC subtypes by targeting EGFR and CD47 surface receptors than single targeting; (2) bypasses the relapse caused by receptor loss during treatment; (3) treats newly diagnosed and drug-resistant TNBCs, and (4) has low off-target toxicity. For the EGFR^−^/CD47^−^ TNBC patients, we can consider targeting alternative surface receptors, such as CD276 (B7-H3) [58,59,60,61], Trop-2 [11,12,13,14,15], or GPR56 [62,63], which have been reported to overexpress in TNBC. In addition, mAb conjugation technology enables tagging multiple antibodies or peptides on EV to target tumors and covering higher portion of patients. Also it is more convenient and flexible to use in cancer treatment as compared to the cell engineering to produce targeted EV.

This study indicated that verrucarin A is involved in the cell cycle downregulation via cyclin D1/p27 signaling pathways in TNBC treatment, which is consistent with the anti-proliferation effect observed in other studies [29]. Further study is needed to analyze other possible anti-TNBC mechanisms such as the pro-survival signaling pathway of Akt/NF-κB/mTOR and mitochondrial depolarization. In future studies, verrucarin A and other therapies with synergetic anti-TNBC mechanisms can be co-delivered by mAb-EV to further improve the anti-tumor efficacy and avoid the reduced efficacy due to drug resistance development in monotherapy.

Multiple nanoparticles have been developed to facilitate drug delivery. For example, the peptide linked liposomes have been applied to deliver doxorubicin to breast cancers [57,64,65]. The polymeric nanoparticles and solid lipid nanoparticles have also been developed to deliver chemotherapies or gene therapies [66,67,68]. As compared to these nanoparticles, EVs have the advantages of high circulation stability and low immune toxicity. This study evaluated one drug (verrucarin A), but mAb-EV could pack and deliver multiple therapies such as chemotherapy, siRNA and gene therapy [29]. Furthermore, the previously reported immune therapeutic functions of EGFR mAb (e.g., antibody-dependent cell cytotoxicity) and CD47 mAb (e.g., reactivation of phagocytosis) could offer additional benefits in TNBC treatment besides directing targeted delivery, but it needs further investigation. 

## 5. Conclusions

The natural extracellular vesicles used in this study had great potential for targeted delivery of a highly potent drug, facilitated with tumor-targeting mAb, for cancer treatment. The natural compound verrucarin A showed high anti-TNBC efficacy with minimal toxicity. Despite the promising results, both the targeted delivery vehicle (mAb-EV) and the therapeutic effect of verrucarin A need full evaluations in preclinical animal models, in terms of pharmacokinetics, pharmacodynamics, biodistribution, toxicology, and anti-TNBC efficacy, in the future. 

## Data Availability

The data presented in this study are contained within the article.
